# Synthesis of Self-Assembled Multifunctional Nanocomposite Catalysts with Highly Stabilized Reactivity and Magnetic Recyclability

**DOI:** 10.1038/srep25459

**Published:** 2016-05-05

**Authors:** Xu Yu, Gong Cheng, Si-Yang Zheng

**Affiliations:** 1 Micro & Nano Integrated Biosystem (MINIBio) Laboratory, Department of Biomedical Engineering, The Pennsylvania State University, University Park, PA 16802, USA

## Abstract

In this paper, a multifunctional Fe_3_O_4_@SiO_2_@PEI-Au/Ag@PDA nanocomposite catalyst with highly stabilized reactivity and magnetic recyclability was synthesized by a self-assembled method. The magnetic Fe_3_O_4_ nanoparticles were coated with a thin layer of the SiO_2_ to obtain a negatively charged surface. Then positively charged poly(ethyleneimine) polymer (PEI) was self-assembled onto the Fe_3_O_4_@SiO_2_ by electrostatic interaction. Next, negatively charged glutathione capped gold nanoparticles (GSH-AuNPs) were electrostatically self-assembled onto the Fe_3_O_4_@SiO_2_@PEI. After that, silver was grown on the surface of the nanocomposite due to the reduction of the dopamine in the alkaline solution. An about 5 nm thick layer of polydopamine (PDA) was observed to form the Fe_3_O_4_@SiO_2_@PEI-Au/Ag@PDA nanocomposite. The Fe_3_O_4_@SiO_2_@PEI-Au/Ag@PDA nanocomposite was carefully characterized by the SEM, TEM, FT-IR, XRD and so on. The Fe_3_O_4_@SiO_2_@PEI-Au/Ag@PDA nanocomposite shows a high saturation magnetization (Ms) of 48.9 emu/g, which allows it to be attracted rapidly to a magnet. The Fe_3_O_4_@SiO_2_@PEI-Au/Ag@PDA nanocomposite was used to catalyze the reduction of *p*-nitrophenol (4-NP) to *p*-aminophenol (4-AP) as a model system. The reaction kinetic constant k was measured to be about 0.56 min^−1^ (R^2^ = 0.974). Furthermore, the as-prepared catalyst can be easily recovered and reused for 8 times, which didn’t show much decrease of the catalytic capability.

Novel metal nanoparticles and nanocomposites have drawn a lot of interest not only due to their unique physical, chemical and biological properties, but also their catalytic activities in many chemical reactions^1^,^2^,^3^,^4^. Recently, more and more attentions are focused on the gold nanoparticles (AuNPs), silver nanoparticles (AgNPs) and their alloys, because of their high catalytic activity, easy fabrication and recyclability^4^,^5^,^6^,^7^. In order to enhance their stability and catalytic activity, a number of materials have been used as the solid supports for the construction of the metal-organic/inorganic materials as hybrid catalysts. For example, graphene^8^, metal-organic framework (MOF)^5^,^9^, mesoporous silica (mSiO_2_)^3^,^10^,^11^, carbon^4^,^12^ and polypyrrole^13^,^14^ have been immobilized the surface of AuNPs. However, tedious centrifugation process is required during the synthesis of the metal-organic/inorganic catalysts. Besides, the recycling of the catalysts is difficult to achieve. Herein, integrating magnetic nanoparticles (especially Fe_3_O_4_ nanoparticles) into the metal–organic/inorganic materials as a magnetically recoverable catalyst provides a promising solution to these problems.

Magnetic particles have many applications in medicine^15^,^16^,^17^, biology^18^,^19^, and other fields^20^,^21^. Their excellent magnetic operability^22^,^23^ greatly favors separation and enrichment of targets^15^ by the external magnetic fields. Some of the magnetic metal-organic/inorganic catalysts were carefully fabricated^6^,^8^,^9^,^24^,^25^. Zheng *et al.* developed an *in situ* growth of the AuNPs on Fe_3_O_4_@SiO_2_ nanocomposite by the Sn^2+^ linkage and reduction^6^. The Sn^2+^ was first absorbed on the surface of the Fe_3_O_4_@SiO_2_ and then used to reduce Au^3+^ by adding sodium formate solution to induce the AuNPs growth. A novel strategy for *in situ* and controlled synthesis of Fe_2_O_3_ on PDA templates through a high temperature thermal decomposition approach was demonstrated by Huang and co-authors^26^. This magnetic nanoparticle was first encapsulated by surfactant-assisted silica followed by template removal by calcination, then functionalized with gold seeds and served as an ideal scaffold for hollow/permeable nanoreactors. This work is impressive, however, it needs high temperature reaction (290 °C) and calcination. Meanwhile, the existing efforts primarily focused on *in situ* synthesis of AuNPs on the supports, in which one typically lacked the degree of control over AuNPs growth as achieved in solution-based synthesis^8^. With the hypothesis that our self-assembly method to construct the functional nanocatalysts might solve these problems, we developed a facile, simple and low-cost method to obtain a novel magnetic–metal nanoparticle hybrid catalyst with enhanced catalytic activity, stability and reusability.

Herein, we proposed to fabricate multifunctional Fe_3_O_4_@SiO_2_@PEI-Au/Ag@PDA nanocomposite catalyst in a simple self-assembled synthesis method (Fig. 1). The magnetic Fe_3_O_4_ nanoparticles were coated with a thin layer of SiO_2_ to obtain a negatively charged surface. Then positively charged PEI polymer was self-assembled onto the Fe_3_O_4_@SiO_2_ surface by the electrostatic interaction. The electrostatic interaction was also adopted to self-assemble the negatively charged AuNPs on the surfaces of PEI coated Fe_3_O_4_@SiO_2_. After that, the dopamine was used to reduce the Ag^+^ to Ag^0^ coated on the AuNPs or the AgNPs on the nanocomposites under an alkaline condition (Tris buffer, pH 8.5). The dopamine is a good reductant, which can be used to synthesize AgNPs via direct reduction of a silver salt solution^27^,^28^,^29^. Meanwhile, the dopamine can autopolymerize in the weak alkaline solution to generate a polydopamine layer, which could coat on almost all the surfaces^30^,^31^,^32^,^33^. Therefore, by the reduction, self-polymerization and the attractive adhesion, the dopamine could be directly used to generate the AgNPs and a layer of nanometer-thick PDA to encapsulate the Fe_3_O_4_@SiO_2_@PEI–Au/Ag nanocomposite. The advantage of this Fe_3_O_4_@SiO_2_@PEI-Au/Ag@PDA nanocomposite prepared by this method is simple, moderate reaction condition and the nanocomposite could be easily separated from the reaction system by using a magnet. The Fe_3_O_4_@SiO_2_@PEI-Au/Ag@PDA nanocomposite shows a high catalytic activity of reducing *p*-nitrophenol (4-NP) to the *p*-aminophenol (4-AP). Furthermore, thanks to the high magnetization and stability, the as-prepared catalyst can be easily recovered by a magnet and reused several times. This high efficient, stable and reusable catalyst might have various applications in catalysis, industrial wastewater treatment, pharmaceutical manufacture, petrochemical production, and food processing.

## Results

### Synthesis of the Fe_3_O_4_@SiO_2_–Au/Ag@PDA nanocomposite by the simple self-assembled method

**Figure 1:**
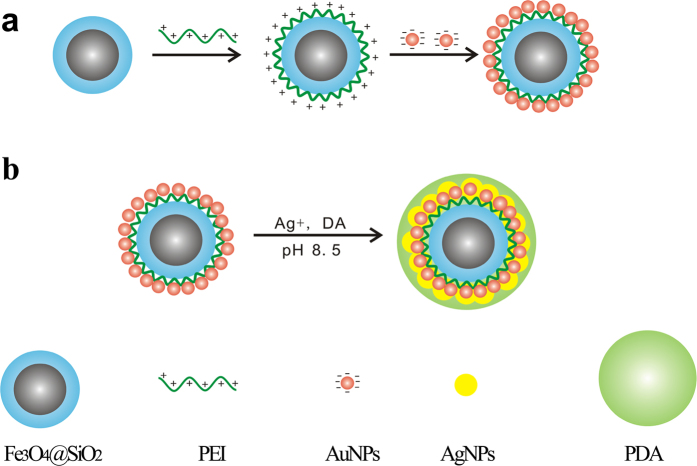
Scheme of synthesis of the Fe_3_O_4_@SiO_2_@PEI–Au/Ag@PDA nanocomposite.

Figure 1 shows the construction strategy of the Fe_3_O_4_@SiO_2_@PEI–Au/Ag@PDA nanocomposite. The electrostatic interactions between the positively charged PEI and negatively charged GSH-AuNPs were used to self-assemble the AuNPs to the Fe_3_O_4_@SiO_2._ The dopamine induced the reduction of the Ag^+^ to Ag^0^ where the Ag was grown either on the AuNPs or on the surfaces of the nanocomposite due to the weak reducing of the dopamine. The Fe_3_O_4_@SiO_2_@PEI–Au/Ag nanocomposite was also encapsulated by the self-polymerization of the dopamine to polydopamine under the alkaline condition. By using this process with moderate conditions, the stable magnetic catalyst could be obtained with a high yield. Our synthesis strategy has many advantages compared to other methods. First, the synthesis approach is simple and easy to repeat. Second, the Fe_3_O_4_@SiO_2_@PEI-Au/Ag@PDA nanocomposite is synthesized under moderate conditions which doesn’t require any high temperature reaction. Third, the dopamine reduced the Ag^+^ to Ag^0^ and coated the Au/AgNPs in a single step under the alkaline condition (Tris buffer, pH 8.5) because at this condition the dopamine can also be oxidized and generate the polydopamine. The coating of a nanometer thick layer of the polydopamine on the Fe_3_O_4_@SiO_2_@PEI-Au/Ag made our catalyst have a good hydrophilic property and excellent stability. During the synthesis process, we find that there is a much stronger adsorption of the Fe_3_O_4_@SiO_2_@AuNPs nanocomposite to the microcentrifuge tube than that of the Fe_3_O_4_@SiO_2_@Au/Ag@PDA (Supporting Information, Fig. S2), which demonstrates that the latter has a better recyclability than the Fe_3_O_4_@SiO_2_@AuNPs nanocomposite.

### Characterization of Fe_3_O_4_@SiO_2_-Au/Ag@PDA nanocomposite

**Figure 2:**
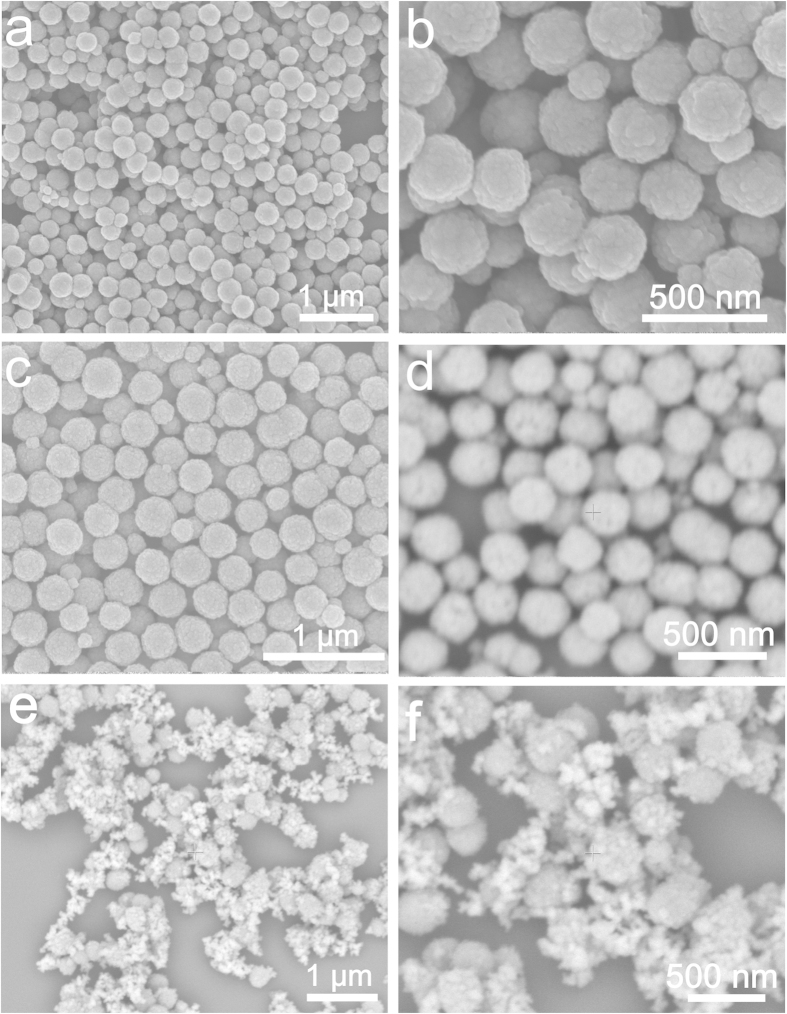
SEM images of the Fe_3_O_4_ NPs (**a**,**b**) low and high magnification of the Fe_3_O_4_ NPs; Fe_3_O_4_@SiO_2_ (**c**) Fe_3_O_4_@SiO_2_ @PEI (**d**) Fe_3_O_4_@SiO_2_@PEI-Au/Ag@PDA (**e**,**f**) low and high magnification of the Fe_3_O_4_@SiO_2_@PEI-Au/Ag@PDA nanocomposite.

**Figure 3:**
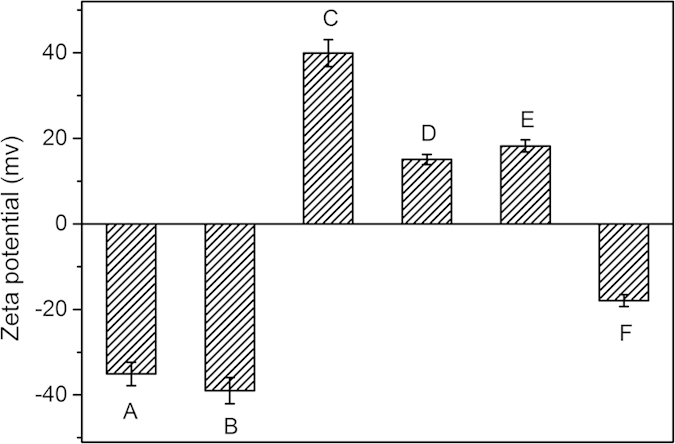
Zeta potentials of the GSH-AuNPs (**A**), Fe_3_O_4_@SiO_2_ (**B**), Fe_3_O_4_@SiO_2_@PEI (**C**), Fe_3_O_4_@SiO_2_@AuNPs (**D**), Fe_3_O_4_@SiO_2_@Au-SH-PEG (**E**) and the Fe_3_O_4_@SiO_2_@PEI-Au/Ag@PDA nanocomposite (**F**) respectively.

The magnetic Fe_3_O_4_ NPs were prepared via a simple hydrothermal reaction based on the high temperature reduction of Fe^3+^ salts with ethylene glycol (EG). As revealed by the SEM in Fig. 2a,b, the magnetic Fe_3_O_4_ NPs have an average diameter of ~300 nm. After coating a thin layer of the SiO_2_ by the sol-gel method in ethanol and NH_3_·H_2_O solution, the Fe_3_O_4_@SiO_2_ didn’t show much difference from the Fe_3_O_4_ NPs in the SEM images (Fig. 2c). The as-prepared Fe_3_O_4_@SiO_2_ showed an excellent monodispersion capability presumably because of its negatively charged surfaces. The zeta potential of the Fe_3_O_4_@SiO_2_ was measured to be about −39.0 mV in water (Fig. 3(B)). The negatively charged Fe_3_O_4_@SiO_2_ NPs could be used for self-assembly with positively charged polyelectrolytes, such as PEI or poly(diallyldimethylammonium chloride), by taking advantage of the electrostatic interaction. In this work, the PEI was adopted and used for coating the Fe_3_O_4_@SiO_2_ NPs. After the PEI coating, the Fe_3_O_4_@SiO_2_@PEI nanocomposite showed less charge in the SEM image because of the polymer on the surface (Fig. 2d), which suggested that PEI was successfully coated on the surfaces of the Fe_3_O_4_@SiO_2_ NPs. The zeta potential of the Fe_3_O_4_@SiO_2_@PEI nanocomposite was measured to be ~ +39.9 mV (Fig. 3(C)) in water, which further confirmed that the positively charged PEI polymer was successfully grafted on the surface of Fe_3_O_4_@SiO_2_ NPs. Because the GSH-capped AuNPs showed a strong negative charge (−35.1 mV, Fig. 3(A)), the AuNPs could be self-assembled on the positively charged Fe_3_O_4_@SiO_2_@PEI NPs through electrostatic interaction. The self-assembly of the GSH-AuNPs on the Fe_3_O_4_@SiO_2_@PEI NPs made the surface potential down to about +15.1 mV (Fig. 3(D)) and the color of the solution changed from gray to black (Data not shown). This is because a lot of the GSH-AuNPs self-assembled onto the Fe_3_O_4_@SiO_2_@PEI nanocomposite. In order to get a good dispersion of the Fe_3_O_4_@SiO_2_@PEI-AuNPs, mPEG-SH was used as a ligand exchange reagent and the PEG modified nanocomposite suspend better in hydrophilic environment. The zeta potential of the Fe_3_O_4_@SiO_2_@PEI-Au-SH-PEG only increased slightly to +18.2 mV (Fig. 3(E)) after the SH-PEG modification, but the hydrophilic solubility significantly improved. After the AgNPs growth by the reduction of Ag^+^ to Ag^0^ and generation a layer of the PDA shell, the zeta potential of the Fe_3_O_4_@SiO_2_@PEI-Au/Ag@PDA nanocomposite changed from +18.2 mV to ~−17.9 mV mainly due to the creation of the PDA shell (Fig. 3(F)). The PDA coating made the nanocomposite disperse better and adsorb less to the microcentrifuge tube than that of the Fe_3_O_4_@SiO_2_@PEI-Au nanocomposite (Supporting Information S3, Fig. S2). Figure 2e,f present the low and high magnification SEM images of the Fe_3_O_4_@SiO_2_@PEI-Au/Ag@PDA nanocomposite. Compared to Fig. 2d, there were many small Au/Ag nanoparticles on the surfaces of the Fe_3_O_4_@SiO_2_@PEI, which demonstrated the successful synthesis of the multifunctional nanocomposite catalyst.

**Figure 4:**
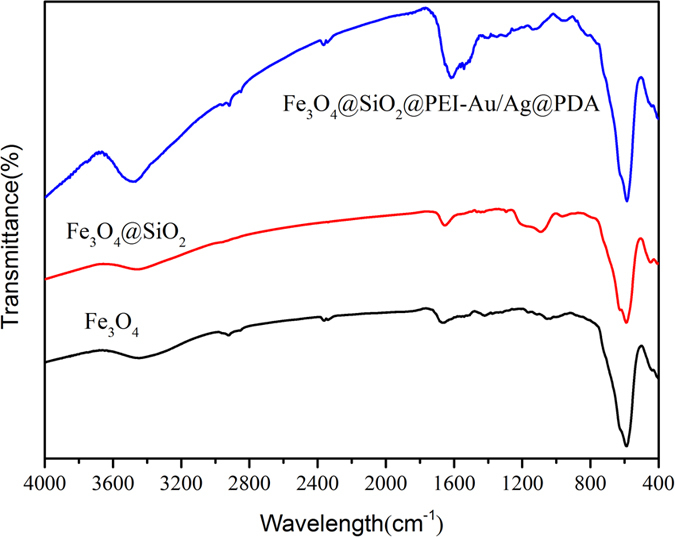
FT-IR characterization of the Fe_3_O_4_, Fe_3_O4@SiO_2_ and Fe_3_O_4_@SiO_2_@PEI-Au/Ag@PDA nanocomposite.

FT-IR spectra were used to study the transformation of the composition of composite of Fe_3_O_4_@SiO_2_, Fe_3_O_4_@SiO_2_ and Fe_3_O_4_@SiO_2_@PEI-Au/Ag@PDA nanocomposite. As shown in Fig. 4, the strong absorption peak at about 585 cm^−1^ in the FT-IR of Fe_3_O_4_ NPs was assigned to the stretching vibration of Fe-O from the Fe_3_O_4_. For the Fe_3_O_4_@SiO_2_ NPs, new and broad absorption bands were observed in the range of 1000–1200 cm^−1^, which could be attributed to the Si-O stretching vibration of the SiO_2_ shell. After generation of the Fe_3_O_4_@SiO_2_@PEI-Au/Ag@PDA nanocomposite, besides the absorption peaks of the Fe_3_O_4_ core and the SiO_2_ shell, strong absorption peaks and some weak peaks were also observed in the range of 1420–1750 cm^−1^ and 2800–3000 cm^−1^ in the FT-IR spectrum. Compared to the Fe_3_O_4_@SiO_2_ NPs these peaks could be attributed to the stretching vibration of the aromatic rings and the C-O stretching of phenol compounds in the PDA shell.

**Figure 5:**
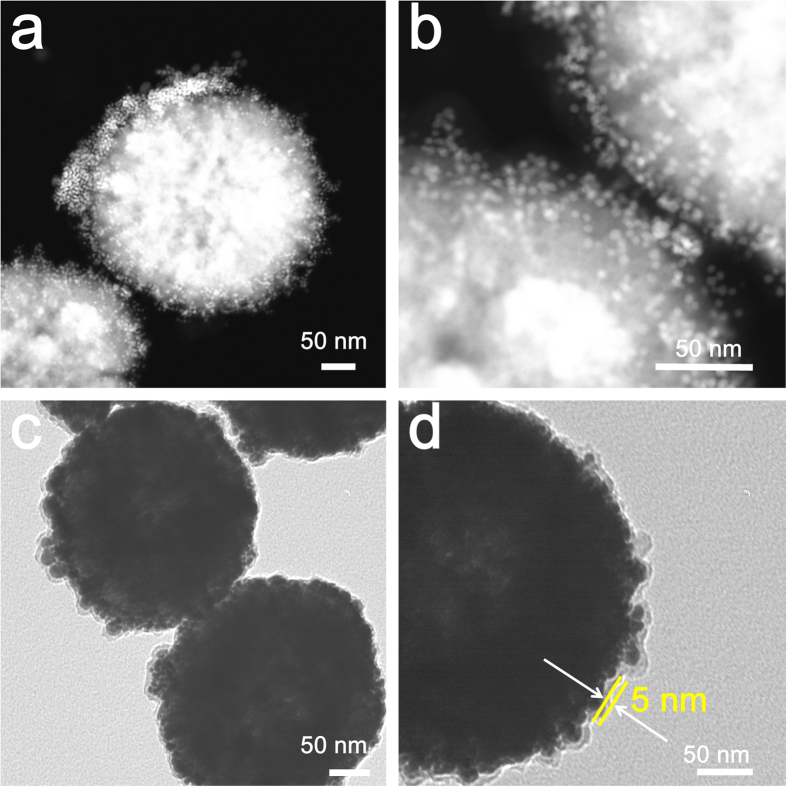
HAADF STEM images of the Fe_3_O_4_@SiO_2_@PEI-Au/Ag@PDA nanocomposite. (**a**,**b**) low and high magnification, respectively; TEM images of the Fe_3_O_4_@SiO_2_@PEI-Au/Ag@PDA (**c**,**d**) low and high magnification of the Fe_3_O_4_@SiO_2_@PEI-Au/Ag@PDA nanocomposite, respectively.

The HAADF STEM and TEM images of the Fe_3_O_4_@SiO_2_@PEI-Au/Ag@PDA nanocomposite were shown in Fig. 5. From the Fig. 5a,b, we can clearly observe the small AuNPs or AgNPs on the surface of the Fe_3_O_4_@SiO_2_@PEI-Au/Ag@PDA nanocomposite. Meanwhile, an about 5 nm PDA layer can be noticed directly in the TEM image (Fig. 5c,d). The thin layer of the PDA made the nanocomposite very stable.

**Figure 6:**
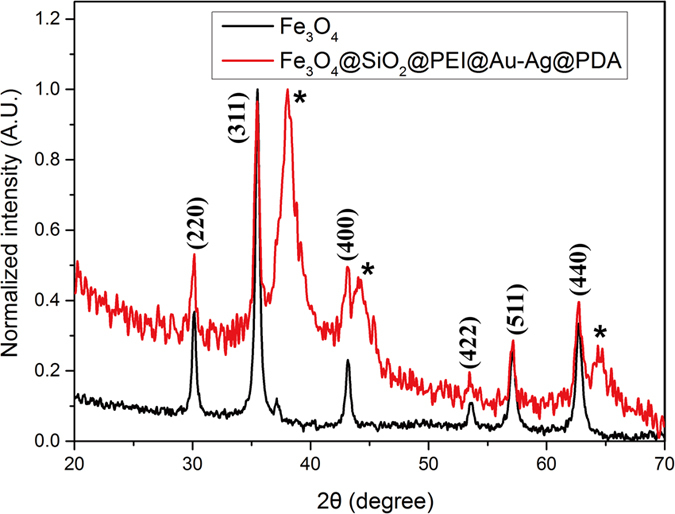
XRD patterns of the Fe_3_O_4_ NPs (black) and the Fe_3_O_4_@SiO_2_@PEI-Au/Ag@PDA nanocomposite (red) (The new diffraction peaks on the Fe_3_O_4_@SiO_2_@PEI-Au/Ag@PDA nanocomposite were labeled with stars (*)).

The crystal structures of the Fe_3_O_4_ NPs and the Fe_3_O_4_@SiO_2_@PEI-Au/Ag@PDA nanocomposite were recorded by the X-ray diffraction (XRD), as shown in Fig. 6. The XRD pattern of the Fe_3_O_4_ shows characteristic peaks at (220), (311), (400), (442), (511) and (440) in agreement to the JCPDS card No. 19-0629. XRD pattern contains no impurity peak indicating the high purity of the Fe_3_O_4_ sample and perfect phase transformation. Obviously, the XRD pattern of the Fe_3_O_4_@SiO_2_@PEI-Au/Ag@PDA nanocomposite has similar diffraction peaks as those of the Fe_3_O_4_ NPs. New diffraction peaks (labeled as ^*^) at 38, 44 and 65 degree were observed, which could be attributed to the diffraction peaks of the AuNPs and AgNPs. However, it is hard to distinguish the diffraction peaks by this method because Au and Ag have very similar diffraction peaks in the XRD pattern.

**Figure 7:**
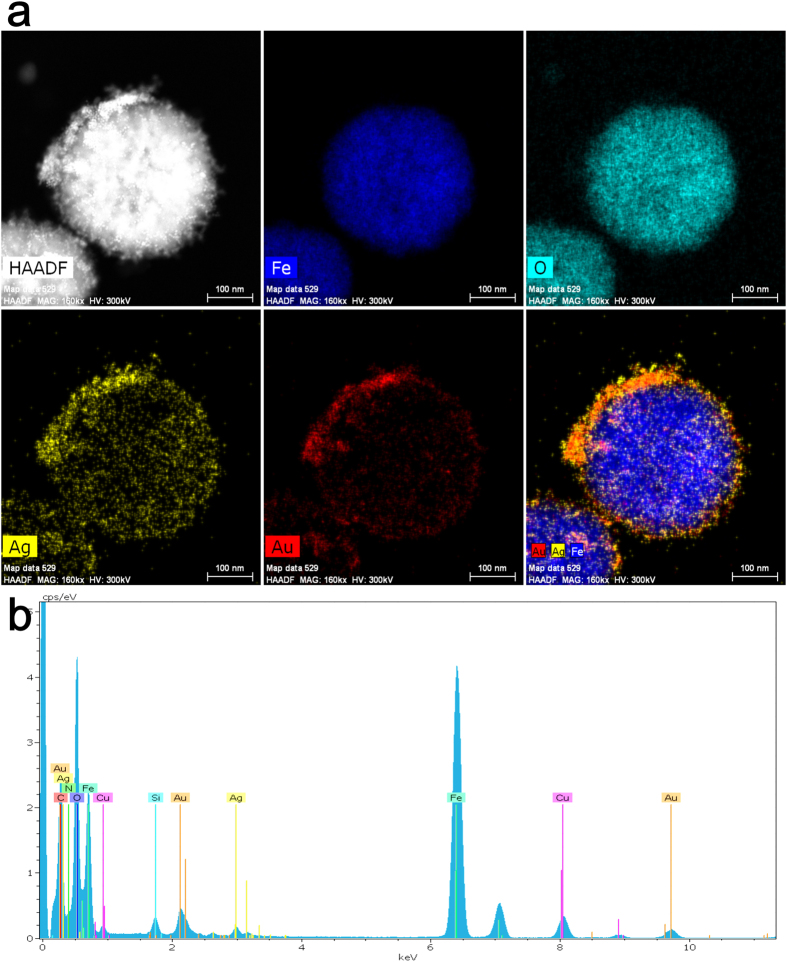
(**a**) HAADF SEM mapping of the elements of the Fe_3_O_4_@SiO_2_@PEI-Au/Ag@PDA nanocomposite; (**b**) The EDX spectrum of the Fe_3_O_4_@SiO_2_@PEI-Au/Ag@PDA nanocomposite. The Cu element comes from the substrate of the Cu grid.

The high angle annular dark field (HAADF) scanning transmission electron microscopy (STEM) was used to map the elements directly on the Fe_3_O_4_@SiO_2_@PEI-Au/Ag@PDA nanocomposite. As shown in the Fig. 7a, we can observe some small particles on the surfaces of the Fe_3_O_4_ in the HAADF STEM images directly, which confirmed that the gold nanoparticles assembled on the Fe_3_O_4_@SiO_2_@PEI nanospheres. The element mapping of the Fe, O, Au and Ag of Fe_3_O_4_@SiO_2_@PEI-Au/Ag@PDA nanocomposite were shown in the Fig. 7a. After merge of the images of the Fe, Au and Ag, they suggest that there was a layer of the Au/Ag around the Fe_3_O_4_ core. From the merge image, we found the positions of Au and Ag elements were highly overlapped. However, it is hard to identify the formation of the Au@Ag core@shell structure by this HAADF mapping. The EDX spectrum of the Fe_3_O_4_@SiO_2_@PEI-Au/Ag@PDA nanocomposite (Fig. 7b) reveals the elements (Fe, O, C, N, Si, Au and Ag) of the composite, confirming the existence of iron oxide, gold, silver, and the PDA organic layer on the nanocomposite. The separated characteristic peak of gold (around 2.1 keV and 9.8 keV) and silver (around 3.1 keV) proved that the AuNPs self-assembled on the nanocomposite and the silver grew onto the nanocomposite.

**Figure 8:**
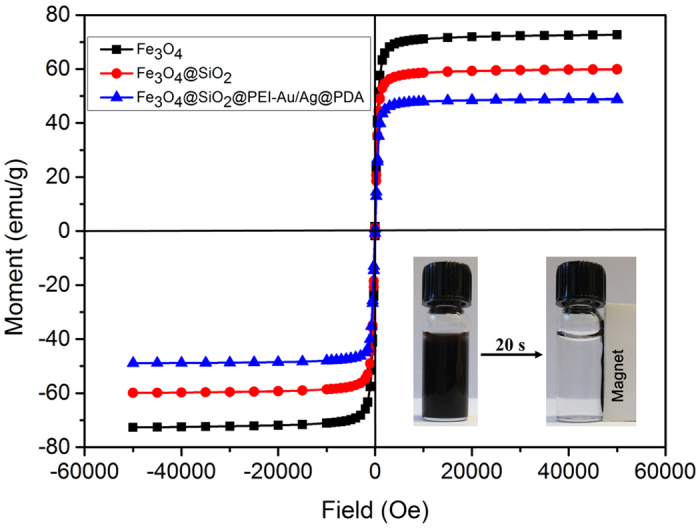
Magnetic hysteresis loops of the Fe_3_O_4_ (black), Fe_3_O_4_@SiO_2_ (red) and the Fe_3_O_4_@SiO_2_@PEI-Au/Ag@PDA nanocomposite (blue) at 300 K. Inset: the photographs showing magnetic Fe_3_O_4_@SiO_2_@PEI-Au/Ag@PDA nanocomposite separated by a magnet.

The magnetic property of nanocomposite is beneficial for its convenient and fast separation, removing the requirements for repeated centrifugation in the practical applications. As shown in Fig. 8, the hysteresis loops of the Fe_3_O_4_, Fe_3_O_4_@SiO_2_ and the Fe_3_O_4_@SiO_2_@PEI-Au/Ag@PDA nanocomposite were measured by a superconducting quantum interface device (SQUID) magnetometer. From the results, the Fe_3_O_4_, Fe_3_O_4_@SiO_2_ and the Fe_3_O_4_@SiO_2_@PEI-Au/Ag@PDA nanocomposite all show strong magnetism characterization at room temperature. The Ms values of the Fe_3_O_4_ and Fe_3_O_4_@SiO_2_ and Fe_3_O_4_@SiO_2_@PEI-Au/Ag@PDA nanocomposite are ~72.6 emu/g, 59.9 emu/g and 48.9 emu/g, respectively. As the introduction of nonmagnetic species, the Ms values of the Fe_3_O_4_@SiO_2_@PEI-Au/Ag@PDA nanocomposite are smaller than that of the Fe_3_O_4_@SiO_2_ and Fe_3_O_4_ nanoparticles. Even after the finally coating with the PDA, the Fe_3_O_4_@SiO_2_@PEI-Au/Ag@PDA nanocomposite still showed a rather high Ms value of 48.9 emu/g, which indicated that the catalyst could be rapidly separated by a common magnet. The Fe_3_O_4_@SiO_2_@PEI-Au/Ag@PDA nanocomposite can be easily dispersed in aqueous solution, and they can be rapidly collected from the mixture within 20s by a permanent magnet. These results showed that the Fe_3_O_4_@SiO_2_@PEI-Au/Ag@PDA nanocomposite can simplify the separation process in practical applications, because of its excellent magnetic response.

### Catalytic performance of Fe_3_O_4_@SiO_2_@PEI-Au/Ag@PDA nanocomposite to convert 4-NP to 4-AP

**Figure 9:**
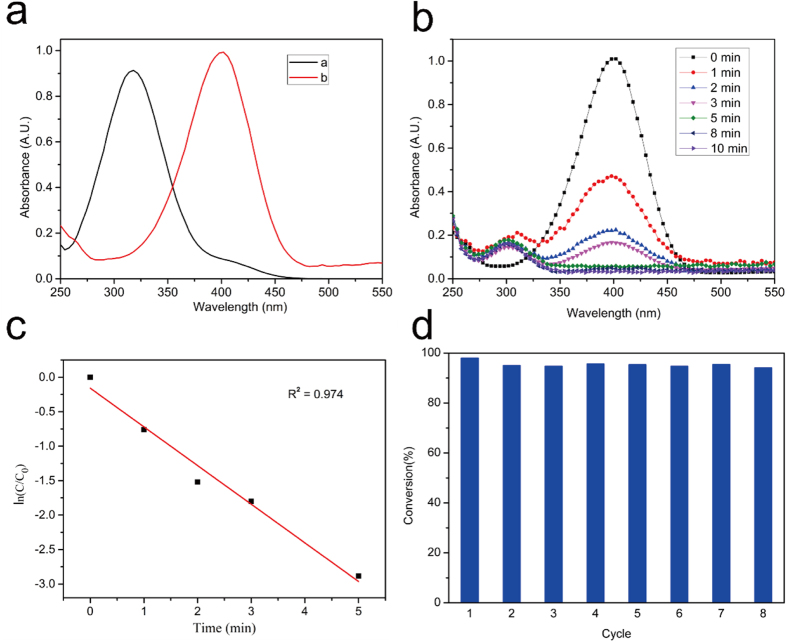
Catalytic performance of Fe_3_O_4_@SiO_2_@PEI-Au/Ag@PDA nanocomposite to convert 4-NP to 4-AP. (**a**) UV–vis spectra of 4-NP (**a**) before and (**b**) after the addition of NaBH_4_; (**b**) Time-dependent UV–vis spectra of the reaction solution in the presence of the Fe_3_O_4_@SiO_2_@PEI-Au/Ag@PDA nanocomposite catalyst; (**c**) Plot of ln (C_t_/C_0_) against the reaction time; (**d**) The recyclability of the Fe_3_O_4_@SiO_2_@PEI-Au/Ag@PDA nanocomposite as the catalyst for the reduction of 4-NP with NaBH_4_.

As a proof-of-concept, the catalytic property of the Fe_3_O_4_@SiO_2_@PEI-Au/Ag@PDA nanocomposite was studied by catalytic reduction of 4-NP to 4-AP with NaBH_4_ (Fig. 9)^28^. After adding NaBH_4_ into the 4-NP, the color of the solution changed from light yellow to bright yellow due to the deprotonation of the 4-NP that made the UV-vis characteristic peak shifted from 317 to 400 nm (Fig. 9A). Without addition of the Fe_3_O_4_@SiO_2_@PEI-Au/Ag@PDA nanocomposite as the catalyst, no obvious change of the UV-vis absorption spectra was observed over time, which demonstrated that the reduction reaction did not proceed without the catalyst. When the catalyst was added, the UV-vis absorption at 400 nm gradually decreased (Fig. 9B), which indicated that the reduction of 4-NP was in progress. Meanwhile, an absorption characteristic peak at 300 nm appeared due to the generation of the 4-AP (Fig. 9B). The conversion of 4-NP to 4-AP completed within 10 min, as the color of the solution transformed from bright yellow into colorless. The catalytic reaction from the 4-NP to 4-AP has been well studied and reported as a first-order reaction^6^,^26^,^34^. The plot of ln (C_t_/C_0_) versus reaction time is linear (Fig. 9C), where C_0_ and C_t_ are the concentrations of 4-NP at time 0 and t measured from the relative absorbance A_t_ and A_0_, respectively. This linear relationship of the reduction reaction matched well with first-order kinetics, and the rate constant k of the reaction with the Fe_3_O_4_@SiO_2_@PEI-Au/Ag@PDA nanocomposite was calculated to be ~0.56 min^−1^ (R^2^ = 0.974). The catalytic performance of the Fe_3_O_4_@SiO_2_@PEI-Au/Ag@PDA nanocomposite to convert the 4-NP to 4-AP is better than that of the self-assembling with only AuNPs or AgNPs into the nanocomposites (Supporting Information S4, Fig. S5). The reaction kinetic constants of Fe_3_O_4_@SiO_2_@PEI-Au@PDA and Fe_3_O_4_@SiO_2_@PEI-Ag@PDA nanocomposites to convert 4-NP to 4-AP are 0.26 min^−1^ and 0.19 min^−1^, respectively (Fig. S3, Fig. S4). Meanwhile, the catalytic activity of our Fe_3_O_4_@SiO_2_@PEI-Au/Ag@PDA nanocomposite was higher than that of most reported catalysts for the same catalytic conversion based on Au or Ag nanoparticles as the catalytic elements (Supporting Information S5, Table S1).

Furthermore, the reusable property of the Fe_3_O_4_@SiO_2_@PEI-Au/Ag@PDA nanocomposite catalyst was investigated by repeated use of the catalyst for several times (Fig. 9D). One significant advantage of this Fe_3_O_4_@SiO_2_@PEI-Au/Ag@PDA nanocomposite is that the recovery process could be easily achieved by simple magnetic separation. The conversion percentage of 4-NP showed little decrease after 8 times cycles, indicating that the Fe_3_O_4_@SiO_2_@PEI-Au/Ag@PDA nanocomposite has an excellent reusability. This can be contributed to the very limited loss of the catalyst during the repeated magnetic separation process, an indication and demonstration of the excellent magnetic property of the Fe_3_O_4_@SiO_2_@PEI-Au/Ag@PDA nanocomposite.

## Conclusions

In summary, we demonstrated a self-assembly approach to fabricate a multifunctional Fe_3_O_4_@SiO_2_@PEI-Au/Ag@PDA nanocomposite catalyst with highly stabilized reactivity and magnetic recyclability. The Fe_3_O_4_@SiO_2_@PEI-Au/Ag@PDA nanocomposite exhibited an excellent catalytic capability. The catalytic reduction of 4-NP to 4-AP obeys a fast first-order kinetics with the reaction constant of 0.56 min^−1^. The Fe_3_O_4_@SiO_2_@PEI-Au/Ag@PDA nanocomposite shows a high saturation magnetization (Ms) of 48.9 emu/g, even after the self-assembly of Au/AgNPs on its surface and an about 5 nm layer of polydopamine coating which is much higher than some previously reported^8,13,26^. The Fe_3_O_4_@SiO_2_@PEI-Au/Ag@PDA NPs can be attracted to a magnet within about 20 s. Therefore, the Fe_3_O_4_@SiO_2_@PEI-Au/Ag@PDA NPs can be easy recyclable and reusable for the next catalytic reaction with a simple magnetic separation and washing process. The high saturation magnetization minimized the loss of the catalyst during the recovery process, which guaranteed a high efficient catalytic performance even after eight times. This simple self-assembled strategy could be used for construction of many magnetic nanocomposite catalysts and the high efficient, stable and reusable catalyst might find many applications in the catalytic field.

## Methods

### Chemicals

Hydrogen tetrachloroaurate (III) hydrate (HAuCl_4_), L-glutathione (GSH), silver nitrate (AgNO_3_) and sodium tetrahydridoborate (NaBH_4_) were purchased from Alfa Aesar. Ferric chloride hexahydrate (FeCl_3_·6H_2_O), ethylene glycol (EG) and sodium acetate (NaAc) were purchased from Alfa Aesar. Tetraethoxysilane (TEOS), poly(ethyleneimine) solution (PEI, 750 KD), dopamine hydrochloride and polyvinylpyrrolidone (PVP, 40 KD) were obtained from Sigma-Aldrich. Methoxy-poly(ethylene glycol)-thiol (mPEG-SH, 2 KD) was purchased from Laysan Bio, Inc. 4-nitrophenol (4-NP) was obtained form EMD Millipore (Billerica, MA, USA). All other chemical reagents used in this work were of analytical grade, obtained from VWR (Radnor, PA, USA), and used without further purification unless otherwise noted.

### Synthesis of Magnetic Fe3O4, Fe_3_O_4_@SiO_2_ NPs and GSH-Capped AuNPs

Fe_3_O_4_ NPs were synthesized as a reported solvothermal approach without much modification^35^. Then the as-prepared Fe_3_O_4_ NPs were coated with a layer of the SiO_2_ according to the method previously reported with slight modification^36^. The detail information of the synthesis of the Fe_3_O_4_ NPs was shown in Supporting Information S1. The size of the Fe_3_O_4_ NPs was about 300 nm. The GSH-capped AuNPs (negative charge) were prepared as our previous report^16^ and the size of the GSH-capped AuNPs is characterized to be about 4 ~ 6 nm from the TEM images (Supporting Information S2, Fig. S1). The process of the synthesis of the AuNPs was described in the Supporting Information S2.

### Electrostatic self-assembly of AuNPs onto the Fe_3_O_4_@SiO_2_ NPs

2.5 mg of Fe_3_O_4_@SiO_2_ nanocomposite was dissolved in 1.8 mL of 0.2 mg/mL PEI solution. After shaking continuously for 0.5 h at room temperature, the as-prepared Fe_3_O_4_@SiO_2_@PEI product was separated using a magnet, rinsed with pure water for three times, and dispersed into 0.5 mL water. Then the negatively charged AuNPs were added into the above solutions under the ultrasonication for 5 min. The solution was kept shaking for 30 min at room temperature. After magnetic separation, the Fe_3_O_4_@SiO_2_@PEI-Au was collected and washed three times with pure water. In order to make sure the maximum electrostatic self-assembly of the AuNPs on the positively charged Fe_3_O_4_@SiO_2_@PEI, the process of adding AuNPs solution, magnetic separation and washing by water was repeated for several times until there were surplus of the AuNPs in the supernatant solution. Finally, the mPEG-SH was added into the as-prepared Fe_3_O_4_@SiO_2_@PEI-Au solution and the system was incubated for 24 h to obtain a good dispersion of the nanocomposite.

### Synthesis of Fe_3_O_4_@SiO_2_@PEI–Au/Ag@PDA Nanocomposite

The Fe_3_O_4_@SiO_2_@PEI–Au/Ag@PDA nanocomposite was prepared by the mussel-inspired coating method. The PEGylated Fe_3_O_4_@SiO_2_@PEI–Au nanocomposite was dispersed in 2 mg/ml of dopamine solution (0.8 mL in 10 mM Tris-HNO_3_ buffer, pH 8.5), followed by slow addition of AgNO_3_ solution (2.5 mM, 0.8 mL). The reaction was allowed to react under shaking for 4 h at room temperature. The obtained Fe_3_O_4_@SiO_2_@PEI–Au/Ag@PDA nanocomposite was collected by a magnet and washed with ultrapure water for 5 times and finally dried in an oven at 60 °C for 12 h.

### Catalytic Reaction of the 4-NP with Fe_3_O_4_@SiO_2_–Au/Ag@PDA nanocomposite

The reduction of 4-NP by NaBH_4_ was chosen as a model reaction for investigating the catalytic performance of the multifunctional Fe_3_O_4_@SiO_2_–Au/Ag@PDA nanocomposite. In a typical procedure, 400 μL of 0.2 M freshly prepared NaBH_4_ was added into the solution containing 12.5 μL of 5 mM 4-NP and 600 μL of H_2_O. Subsequently, 50 μL of 2 mg/mL catalyst was added into the above solution and the reaction started immediately. The color change of the solution from bright yellow to colorless was observed during the reaction process. The UV-vis absorption spectra of the solution were recorded to monitor the progress of the reaction by using a microplate reader with the scanning range from 250 nm to 550 nm. The kinetic rate constants of the reduction process were determined by measuring the change in absorbance at 400 nm as a function of time. In order to study the recyclability of the catalyst, the used Fe_3_O_4_@SiO_2_@PEI–Au/Ag@PDA nanocomposite was harvested from the reaction mixture by a magnet at the end of each run, washed with pure water by three times, then re-dispersed in 50 μL water and added in a fresh reaction solution. After reaction for 10 min, the Fe_3_O_4_@SiO_2_–Au/Ag@PDA nanocomposite was removed and the supernatant solution was measured to evaluate the recyclability of the catalysts. In total, this procedure was repeated 8 times.

### Characterization

Scanning electron microscopy (SEM) images were obtained on a field-emission scanning electron microscope (NanoSEM 630, NOVA). Transmission electron microscopy (TEM) images were conducted with a Philips-420 microscope at an acceleration voltage of 120 kV. The high angle annular dark field (HAADF) images was obtained by a scanning transmission electron microscopy TITAN (FEI Titan3 G2). The zeta potential was measured by the Malvern Zetasizer ZS. Fourier transform infrared spectra were obtained by a Bruker Vertex V70 FT-IR spectrometer scanned from 400 to 4000 cm^−1^. The powder X-ray diffraction (PXRD) patterns were performed by a PANalytical Empyrean X-ray powder diffractometer with Cu target (45 kV, 40 mA) from 20 to 70 degrees. The magnetization measurement was collected by a superconducting quantum interface device (SQUID) magnetometer at 300 K.

## Additional Information

**How to cite this article**: Yu, X. *et al.* Synthesis of Self-Assembled Multifunctional Nanocomposite Catalysts with Highly Stabilized Reactivity and Magnetic Recyclability. *Sci. Rep.* **6**, 25459; doi: 10.1038/srep25459 (2016).

## Supplementary Material

Supplementary Information
