# Supplementary material for: Synthesis of Self-Assembled Multifunctional Nanocomposite Catalysts with Highly Stabilized Reactivity and Magnetic Recyclability
Source: Sci Rep. 2016 May 5;6:25459. doi: 10.1038/srep25459 (PMC4857104; doi:10.1038/srep25459)
Supplement: Supplementary Information [file srep25459-s1.pdf]

Synthesis of Self-Assembled Multifunctional Nanocomposite Catalysts with Highly Stabilized Reactivity and Magnetic Recyclability

Xu Yu, Gong Cheng and Si-Yang Zheng*

Micro & Nano Integrated Biosystem (MINIBio) Laboratory, Department of Biomedical Engineering, The Pennsylvania State University, University Park, PA 16802, USA.

*Corresponding Author: Si-Yang Zheng, N-238 Millennium Science Complex, University Park, PA 16802 USA, Tel: (814) 865-8090, Fax: (814) 863-0490, Email: sxz10@psu.edu

KEYWORDS: Synthesis, Self-assembly, Multifunctional, Magnetic Nanocomposites, Catalyst, Polydopamine

S1. Synthesis of Magnetic Fe_3O_4 NPs and Fe_3O_4@SiO_2 NPs

Fe_3O_4 NPs and the Fe_3O_4@SiO_2 NPs were synthesized as previously reported method with slight modification[1, 2]. The magnetic Fe_3O_4 NPs were prepared through a solvothermal reaction[1]. Briefly, 1.35 g of $FeCl_3 \cdot 6H_2O$ and 3.6 g of sodium acetate were dissolved in 50 mL of ethylene glycol under magnetic stirring for 1 h. After that, the obtained yellow solution was transferred to a Teflonlined stainless steel autoclave and sealed to heat at 200 °C for 8 hours. The autoclave was cooled to room temperature and the obtained black magnetite particles were washed with ethanol and water for several times and dried at 60 °C for 12 h. The general procedure to coat Fe_3O_4 NPs with silica is consisted of two steps: adsorption of the PVP and the growth of the silica shell after transfer of the particles to ethanol[2]. Typically, 30 mg of the Fe_3O_4 NPs were

treated with 0.1 M HCl by ultrasonication for 15 min. Then the Fe_3O_4 NPs were washed by ultrapure water and ethanol for 5 times, respectively. The Fe_3O_4 NPs were dispersed in a 10 mL of 20 mg/mL of PVP ethanol solution and incubated at room temperature under continuous magnetic stirring for 24 hours in order to guarantee that adsorption of the PVP was complete. After that, 330 μL of ammonia solution (28 %) was added to the solution, then 1 mL of TEOS solution (10 % vol TEOS in ethanol) was added. The reaction was allowed to proceed at room temperature for about 12 h. Then another 1 mL of TEOS solution (10% vol TEOS in ethanol) was added and the reaction continued for another 12 h to allow the growth of a thin silica shell. The solid product was collected by using an external magnet and rinsed with ultrapure water for several times.

S2. Synthesis of GSH-Capped AuNPs

The GSH-capped AuNPs (negative charged) were prepared as our previous report[3]. Briefly, 1 mL of a 1% $HAuCl_4$ aqueous solution was mixed with 7.8 mL of 45 mg GSH in aqueous solution under magnetic stirring. The pH value of the resulting mixture was adjusted to 8.0 using 10 M NaOH. Then, 4.5 mL of freshly prepared $NaBH_4$ aqueous solution (2 mg/mL) was quickly added into the above solution with vigorous stirring. The mixture was allowed to react for 12 h at room temperature. The product was then purified by Amicon Centrifugal Filter Unit (MWCO 30 kDa) to remove the free reagents and then dispersed in ultrapure water and stored at 4 °C until further experiments.

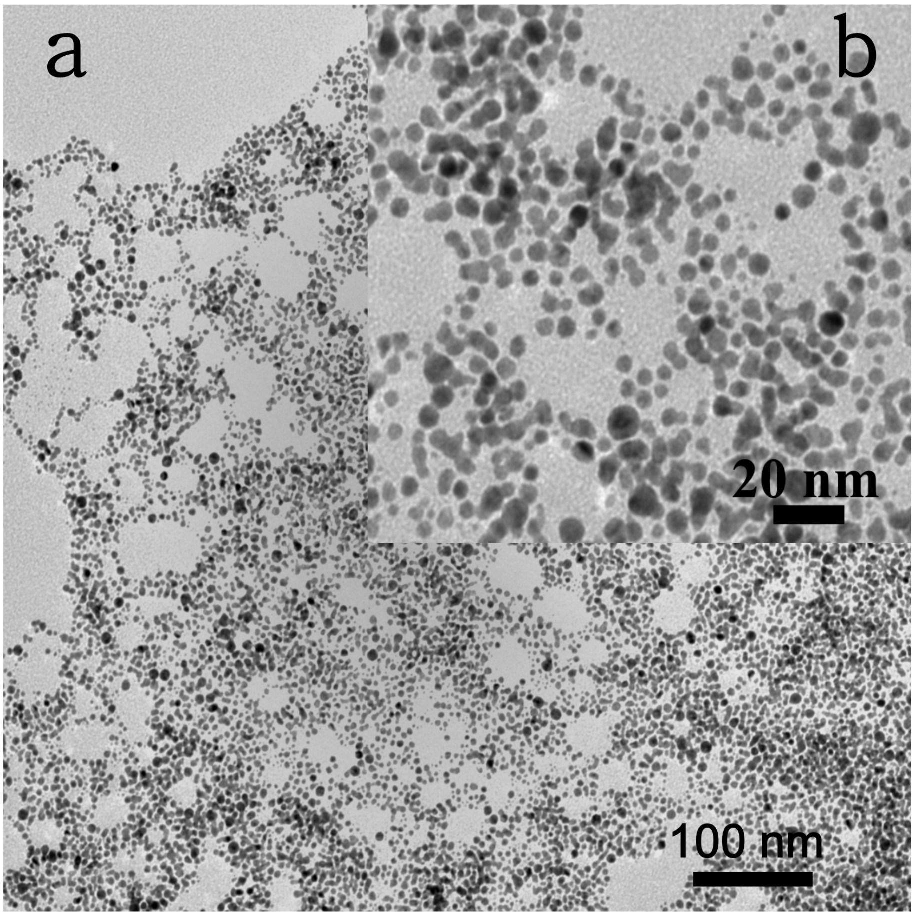

Figure. S1 TEM image of the AuNPs-GSH (a). Inset: High magnification of the TEM image of the AuNPs-GSH (b).

S3. Comparison of the adsorption of the nanocomposites with or without the PDA coating

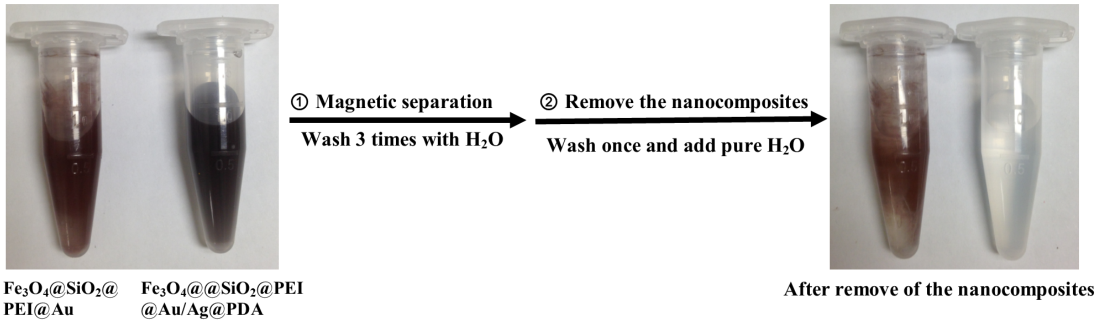

Figure. S2 Comparison of the adsorption of the $Fe_3O_4@SiO_2@Au/Ag@PDA$ nanocomposite and the $Fe_3O_4@SiO_2@AuNPs$ nanocomposite to the microcentrifuge tube.

S4. Comparison of the catalytic performance of nanocomposites Fe_3O_4@SiO_2@PEI-Au@PDA, Fe_3O_4@SiO_2@PEI-Ag@PDA and Fe_3O_4@SiO_2@PEI-Au/Ag@PDA

The GSH-capped AgNPs were prepared as previous report[4]. The self-assembly process to synthesize the Fe_3O_4@SiO_2@PEI-Au@PDA nanocomposite and Fe_3O_4@SiO_2@PEI-Ag@PDA nanocomposite was similar to that of the Fe_3O_4@SiO_2@PEI-Au/Ag@PDA nanocomposite. The only difference was not adding the $AgNO_3$ solution into the Fe_3O_4@SiO_2@PEI-Au or Fe_3O_4@SiO_2@PEI-Ag when the PDA was introduced in the last step. The catalytic performance of the Fe_3O_4@SiO_2@PEI-Au@PDA and Fe_3O_4@SiO_2@PEI-Ag@PDA nanocomposites was tested at the same condition as that of the Fe_3O_4@SiO_2@PEI-Au/Ag@PDA nanocomposite. The catalytic performances of Fe_3O_4@SiO_2@PEI-Au@PDA and Fe_3O_4@SiO_2@PEI-Ag@PDA nanocomposite to convert 4-NP to 4-AP are shown in **Figure S3** and **Figure S4.** The reaction constants were measured as 0.26 min^{-1} and 0.19 min^{-1} respectively. **Figure S5** depicts the comparison of the plots of $\ln(C_t/C_0)$ versus the reaction time of the Fe_3O_4@SiO_2@PEI-Au@PDA, Fe_3O_4@SiO_2@PEI-Ag@PDA and Fe_3O_4@SiO_2@PEI-Au/Ag@PDA respectively. The results demonstrate that the catalytic performance of the Fe_3O_4@SiO_2@PEI-Au/Ag@PDA is significantly better than that of the Fe_3O_4@SiO_2@PEI-Au@PDA and Fe_3O_4@SiO_2@PEI-Ag@PDA.

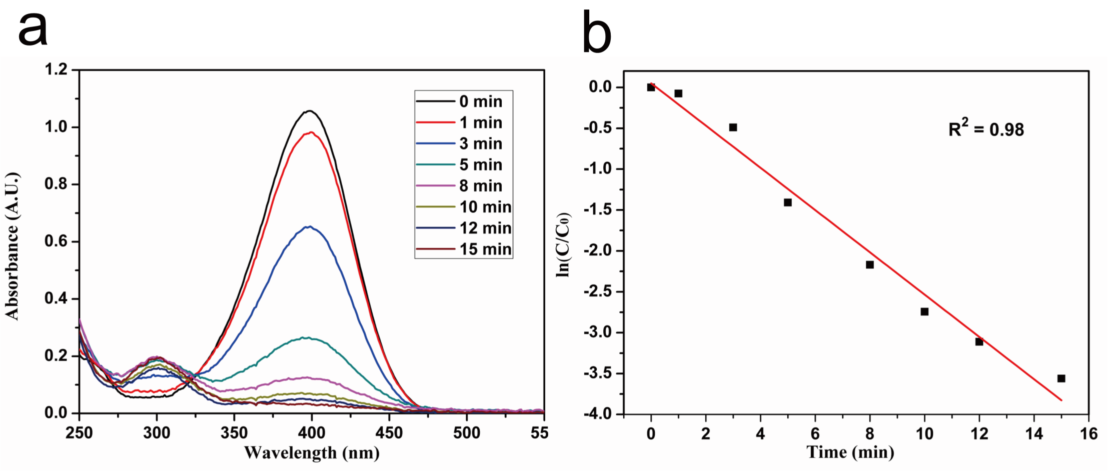

Figure S3. Catalytic performance of the Fe_3O_4@SiO_2@PEI-Au@PDA nanocomposite to convert 4-NP to 4-AP. (a) Time-dependent UV–vis spectra of the reaction in the presence of the Fe_3O_4@SiO_2@PEI-Au@PDA nanocomposite catalyst; (b) Plot of $\ln(C_t/C_0)$ versus the reaction time. The catalytic reduction of 4-NP to 4-AP Fe_3O_4@SiO_2@PEI-Au@PDA obeys fast first-order kinetics with the measured reaction constant of 0.26 min^{-1}.

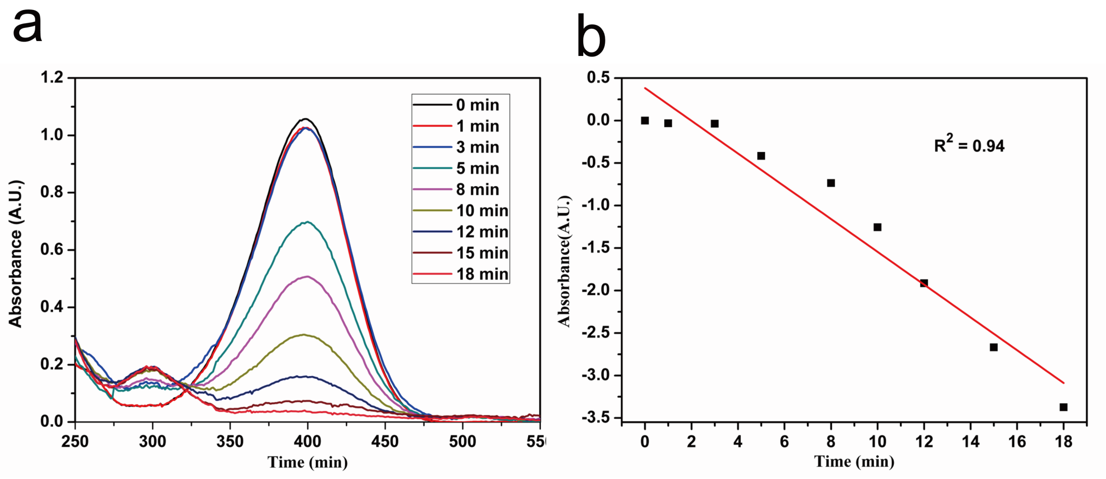

Figure S4. Catalytic performance of Fe_3O_4@SiO_2@PEI-Ag@PDA nanocomposite to convert 4-NP to 4-AP. (a) Time-dependent UV–vis spectra of the reaction in the presence of the Fe_3O_4@SiO_2@PEI-Ag@PDA nanocomposite catalyst; (b) Plot of $\ln(C_t/C_0)$ versus the reaction

time. The catalytic reduction of 4-NP to 4-AP Fe_3O_4@SiO_2@PEI-Ag@PDA obeys fast first-order kinetics with the measured reaction constant of 0.19 min^{-1}.

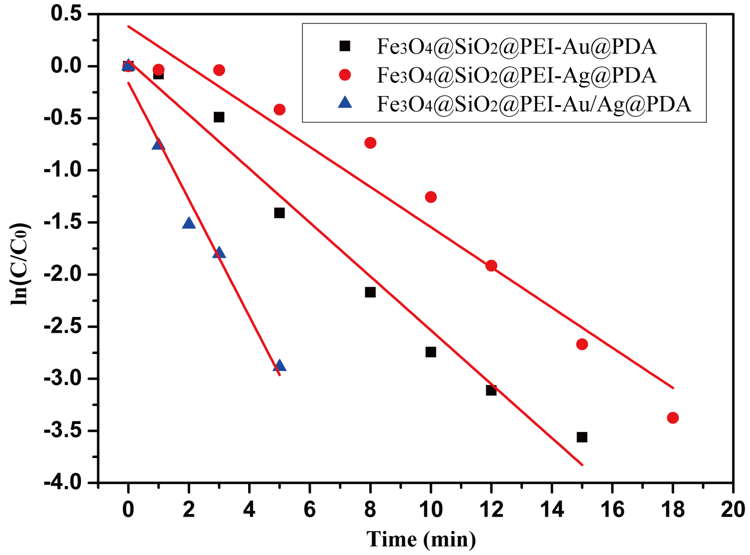

Figure S5. Comparison of the catalytic performances of Fe_3O_4@SiO_2@PEI-Au@PDA nanocomposite, Fe_3O_4@SiO_2@PEI-Ag@PDA nanocomposite and Fe_3O_4@SiO_2@PEI-Au/Ag@PDA nanocomposite to convert 4-NP to 4-AP.

S5. Comparison of the magnetic property and catalytic activity of our catalyst with some catalysts reported in the literature.

Table S1 Comparison of the magnetic property and catalytic activity for the reduction of 4-NP to 4-AP between Fe_3O_4@SiO_2@PEI-Au/Ag@PDA and some catalysts reported in the literature.

Catalyst	Catalyst element	Saturated magnetization value (emu/g)	k (min^{-1})	Reference
Au@hollow silica	Au	/	0.039-0.06	5
Au@meso-SiO_2	Au	/	0.08	6
Au/GO	Au	/	0.188	7
Au@Ag@MOF	Au/ Ag	/	0.298	8
Au@C	Au	/	0.48	9
Fe_2O_3@Au@SiO_2	Au	15.1	0.38	10
γ-Fe_2O_3/Au/mSiO_2	Au	8.4	0.4	11
Fe_3O_4@SiO_2-Au@mSiO_2	Au	18.6	0.20-0.35	12
Fe_3O4@SiO_2@Ag	Ag	38.58	0.13-0.83	13
Fe_3O_4@PDA@RGO@Au	Au	40.79	0.725	14
Fe_3O_4@SiO_2@PEI-Au/Ag@PDA	Au/Ag	48.9	0.56	this work

Compared with the literature (Table S1), this synthesized Fe_3O_4@PEI@Au/Ag@PDA catalyst exhibited comparable or much better catalytic activity for 4-NP reduction.

References

1. Deng, Y.; Qi, D.; Deng, C.; Zhang, X.; Zhao, D. Superparamagnetic high-magnetization microspheres with an Fe_3O_4@SiO_2 core and perpendicularly aligned mesoporous SiO_2 shell for removal of microcystins. *J Am Chem Soc* **130**, 28-29 (2008).

2. Graf, C.; Vossen, D. L. J.; Imhof, A.; van Blaaderen, A. A general method to coat colloidal particles with silica. *Langmuir* **19**, 6693-6700 (2003).

3. Yu, X.; Zhang, Z. L.; Zheng, S. Y. Highly sensitive DNA detection using cascade amplification strategy based on hybridization chain reaction and enzyme-induced metallization. *Biosens Bioelectron* **66**, 520-526 (2015).

4. Li, H.; Cui, Z.; Han, C.; Glutathione-stabilized silver nanoparticles as colorimetric sensor for Ni^{2+} ion, *Sensor Actuat B-Chem* **143**, 87-92 (2009).

5. Wu, S. H. et al. Catalytic nano-rattle of Au@hollow silica: towards a poison-resistant nanocatalyst. *J. Mater. Chem.* **21**, 789-794 (2011).

6. Chen, J.; Xue, Z.; Feng, S.; Tu, B.; Zhao, D. Synthesis of mesoporous silica hollow nanospheres with multiple gold cores and catalytic activity. *J. Colloid Interface Sci.* **429**, 62-67 (2014).

7. Zhang, Y. et al; In situ green synthesis of Au nanostructures on graphene oxide and their application for catalytic reduction of 4-nitrophenol. *Catal. Sci. Technol.* **1**, 1142-1144 (2011).

8. Jiang, H. L.; Akita, T.; Ishida, T.; Haruta, M.; Xu, Q. Synergistic catalysis of Au@Ag core-shell nanoparticles stabilized on metal-organic framework. *J Am Chem Soc* **133**, 1304-1306 (2011).

9. Liu, R.; Qu, F.; Guo, Y.; Yao, N.; Priestley, R. D. Au@carbon yolk-shell nanostructures via one-step core-shell-shell template. *Chem Commun* **50**, 478-480 (2014).

10. Huang, L. et al. Iron oxide nanoparticle layer templated by polydopamine spheres: a novel scaffold toward hollow-mesoporous magnetic nanoreactors. *Nanoscale* **7**, 806-813, (2015).

11. Ren, L.; Teng, C.; Zhu, L.; He, J.; Wang, Y. et al. Preparation of uniform magnetic recoverable catalyst microspheres with hierarchically mesoporous structure by using porous polymer microsphere template. *Nanoscale Res Lett* **9**, 163 (2014).

12. Deng, Y. et al. Multifunctional mesoporous composite microspheres with well-designed nanostructure: a highly integrated catalyst system. *J Am Chem Soc* **132**, 8466-8473 (2010).

13. Du, X.; He, J.; Zhu, J.; Sun, L.; An, S. Ag-deposited silica-coated Fe_3O_4 magnetic nanoparticles catalyzed reduction of p-nitrophenol. *Appl. Surf. Sci.* **258**, 2717-2723 (2012).

14. Zeng, T.; Zhang, X. L.; Ma, Y. R.; Niu, H. Y.; Cai, Y. Q. A novel Fe_3O_4–graphene–Au multifunctional nanocomposite: green synthesis and catalytic application. *J Mater Chem* **22**, 18658-18663 (2012).
